# Palmitoylethanolamide Modulation of Microglia Activation: Characterization of Mechanisms of Action and Implication for Its Neuroprotective Effects

**DOI:** 10.3390/ijms22063054

**Published:** 2021-03-17

**Authors:** Alessia D’Aloia, Laura Molteni, Francesca Gullo, Elena Bresciani, Valentina Artusa, Laura Rizzi, Michela Ceriani, Ramona Meanti, Marzia Lecchi, Silvia Coco, Barbara Costa, Antonio Torsello

**Affiliations:** 1 Department of Biotechnology and Biosciences, University of Milano-Bicocca, 20126 Milano, Italy; alessia.daloia@unimib.it (A.D.); francesca.gullo@unimib.it (F.G.); v.artusa@campus.unimib.it (V.A.); michela.ceriani@unimib.it (M.C.); marzia.lecchi1@unimib.it (M.L.); 2 School of Medicine and Surgery, University of Milano-Bicocca, 20900 Monza, Italy; laura.molteni@unimib.it (L.M.); elena.bresciani@unimib.it (E.B.); laura.rizzi@unimib.it (L.R.); r.meanti@campus.unimib.it (R.M.); silvia.coco@unimib.it (S.C.)

**Keywords:** palmitoylethanolamide, microglia, morphotypes, neuroinflammation, cytokines, cannabinoid receptor, LPS, electrophysiology

## Abstract

Palmitoylethanolamide (PEA) is an endogenous lipid produced on demand by neurons and glial cells that displays neuroprotective properties. It is well known that inflammation and neuronal damage are strictly related processes and that microglia play a pivotal role in their regulation. The aim of the present work was to assess whether PEA could exert its neuroprotective and anti-inflammatory effects through the modulation of microglia reactive phenotypes. In N9 microglial cells, the pre-incubation with PEA blunted the increase of M1 pro-inflammatory markers induced by lipopolysaccharide (LPS), concomitantly increasing those M2 anti-inflammatory markers. Images of microglial cells were processed to obtain a set of morphological parameters that highlighted the ability of PEA to inhibit the LPS-induced M1 polarization and suggested that PEA might induce the anti-inflammatory M2a phenotype. Functionally, PEA prevented Ca^2+^ transients in both N9 cells and primary microglia and antagonized the neuronal hyperexcitability induced by LPS, as revealed by multi-electrode array (MEA) measurements on primary cortical cultures of neurons, microglia, and astrocyte. Finally, the investigation of the molecular pathway indicated that PEA effects are not mediated by toll-like receptor 4 (TLR4); on the contrary, a partial involvement of cannabinoid type 2 receptor (CB2R) was shown by using a selective receptor inverse agonist.

## 1. Introduction

Microglia are resident macrophage-like cells of the central nervous system (CNS). For a long time, they were considered to be quiescent in healthy conditions, becoming activated in pathological situations. However, it is now accepted that microglia in “resting” state participate in fundamental aspects of neuronal homeostasis and act to surveil the CNS; this surveillant/non-polarized phenotype is also known as M0 [1]. Upon injury, microglia become activated, switching between pro-inflammatory (M1) phenotype, producing cytokines such as tumor necrosis factor alpha (TNF-α) and interleukin-1 beta (IL-1β), and anti-inflammatory/neuroprotective (M2) phenotype, expressing interleukin-10 (IL-10). M2 microglia are further characterized into sub-classifications: anti-inflammatory (M2a), inflammation modulatory (M2b), and immunosuppressive (M2c), even if microglia phenotype is not absolute since microglial cells show high plasticity [2]. The presence of multiple activation phenotypes for microglia is a relatively new concept; therefore, the roles they play are still not fully characterized. However, it is well established that during chronic neuroinflammation, which underlies many neurodegenerative diseases, microglial cells are continually activated by proinflammatory stimuli. These chronically activated microglia will continue to produce inflammatory cytokines and reactive oxygen/nitrogen species, which lead to neuronal death [3]. This makes microglia modulation an attractive therapeutic tool in pathological conditions where detrimental polarization may contribute to disease. Thus, the identification of compounds acting as microglia modulators, i.e., able not only to prevent the detrimental proinflammatory phenotype, but also to promote the beneficial alternative phenotype, can be a therapeutic tool to mitigate various neuro-pathologies that share chronic microglia hyper-reactivity (Alzheimer’s disease, multiple sclerosis, stroke).

Palmitoylethanolamide (PEA) belongs to the class of the *N*-acylethanolamine and is an endogenous lipid potentially useful in a wide range of therapeutic areas. PEA is produced on-demand by neurons and glial cells in the CNS and is involved in the endogenous neuroprotective mechanisms that are activated following tissue damage or inflammation [4], suggesting that its exogenous contribution may favor the process of resolution of inflammation and the restoration of tissue homeostasis. Many studies have demonstrated that the anti-inflammatory property of PEA is accomplished through the inhibition of the release of pro-inflammatory molecules from mast cells and macrophages [5,6]. More recently, the PEA ability to induce microglia changes associated with increased migration and phagocytic activity has been reported [7]. Even though some studies have suggested that PEA can exert its neuroprotective effect through the microglia modulation, no extensive characterization has been done. The aim of this study was to assess whether PEA could control microglia polarization in order to furnish further insights on endogenous PEA protective and pro-homeostatic mechanism and to suggest its exogenous administration as a useful pharmacological tool for controlling neuroinflammatory conditions.

In this work, we studied the effect of PEA on lipopolysaccharide (LPS)-induced microglia activation by (1) evaluating the markers defining microglia phenotypes; (2) performing a morphometric analysis, which is considered as a valuable method to better understand form and function relationships in microglia; (3) analyzing the microglial Ca^2+^ signals as a rapid functional tool of microglia activation; (4) assessing neuron–glia cross-talk through the multi-electrode array (MEA) recording method; and (5) studying the possible receptor involved.

## 2. Results

### 2.1. PEA Pre-Treatment Counteracts M1 Microglia Polarization Induced by LPS Treatment

**Figure 1:**
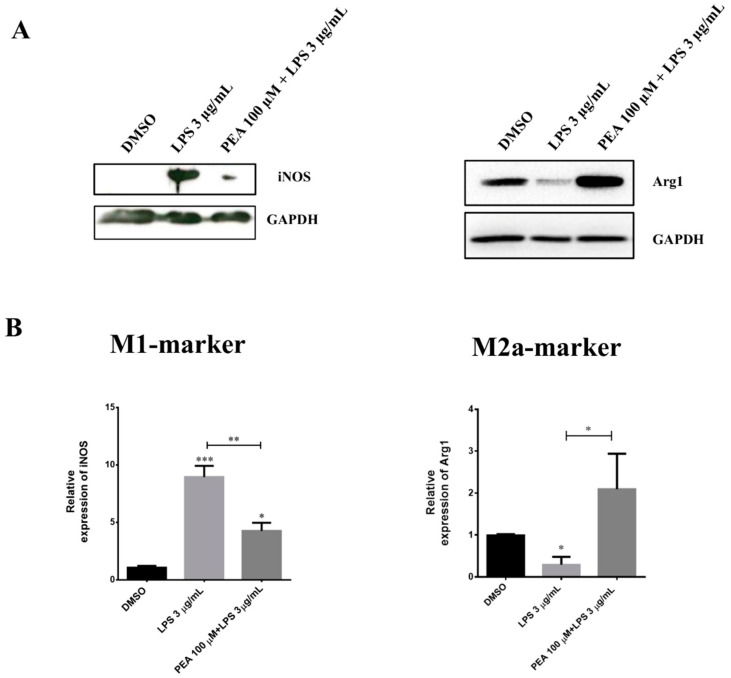
Effect of palmitoylethanolamide (PEA) on M1/M2 markers in lipopolysaccharide (LPS)-stimulated N9 cells. Cells were incubated with 100 µM PEA at 1 h before treatment with 3 µg/mL lipopolysaccharide (LPS) for 6 h. Total protein extract from N9 cells were separated on SDS-PAGE and transferred to a polyvinylidene difluoride (PVDF) membrane; membranes were probed with anti-iNOS and anti-Arg1. GAPDH was used to normalize sample loading. Panel (**A**): A representative Western blot is shown. Panel (**B**): Histograms relative to the quantification of bands of inducible nitric oxide synthase (iNOS) and arginase-1 (Arg1) after dimethyl sulfoxide (DMSO), LPS, and PEA + LPS treatments. Data are shown as the mean ± S.E.M. (*n* = three independent experiments). Differences among groups were tested for significance by the one-way analysis of variance (ANOVA) followed by Tukey post hoc test (for iNOS) or by Kruskal–Wallis test followed by Dunn’s multiple comparisons test (for Arg1). * *p* < 0.05, ** *p* < 0.01, *** *p* < 0.001.

The effects of PEA were first assessed on the pro-inflammatory activity of LPS in N9 microglia cells. Dose-response and time-course experiments were first performed to select the optimal concentration of PEA and incubation time to be used. The results of these preliminary experiments indicated that 100 µM PEA, administered 1 h before LPS treatment, was the best condition to observe reduction of the effects induced by LPS. These conditions were maintained in the subsequent experiments. The activation states of microglia were assessed by measuring the levels of inducible nitric oxide synthase (iNOS), a M1 marker, and those of arginase-1 (Arg1), a M2a marker, in N9 cells incubated for 6 h with LPS. Levels of iNOS and Arg1 were measured by Western blot. LPS significantly increased iNOS levels and concomitantly inhibited those of Arg1 compared to controls (Figure 1A). The LPS-induced iNOS increase was significantly blunted in cells pre-treated with 100 µM PEA for 1 h (Figure 1B). Of particular interest is that PEA pre-treatment antagonized LPS-induced Arg1 reduction and restored it to control values (Figure 1B). Given that the increase of Arg1 is considered a specific marker of the subtype anti-inflammatory M2a phenotype, these data highlight the ability of PEA to inhibit the LPS-induced M1 polarization of microglia and suggest that PEA might induce a M2a phenotype upon injury. To further strengthen these hypotheses, N9 cells were treated with 100 µM PEA alone for 1 h (Figure S1), and results demonstrated a trend toward an increase in Arg1 levels, even if it did not reach statistical significance.

**Figure 2:**
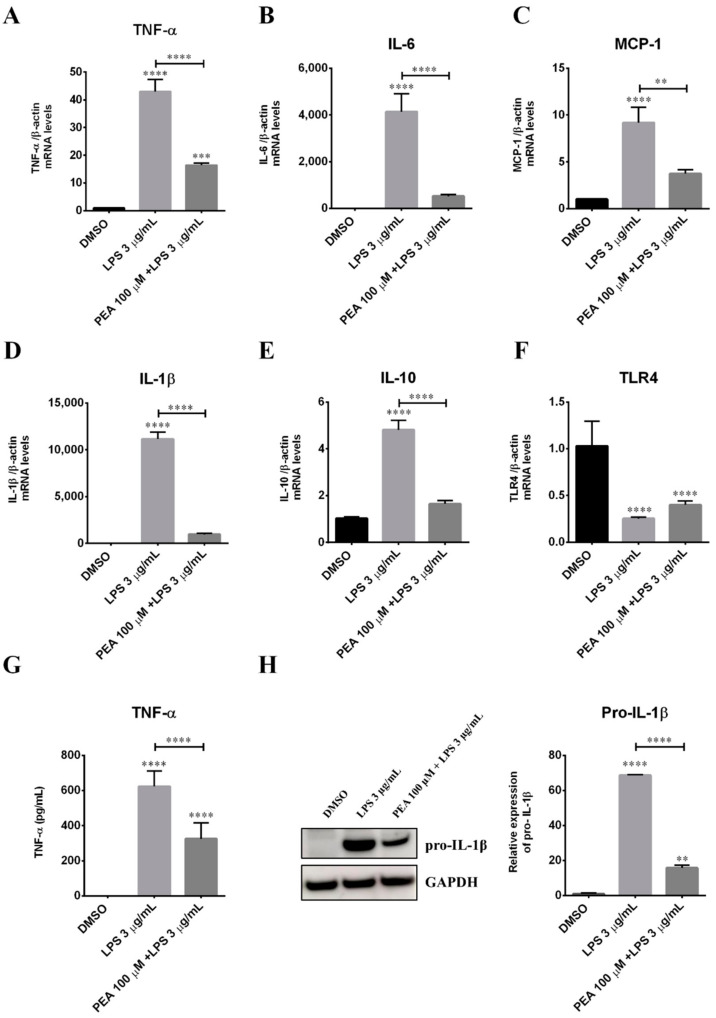
Effect of PEA on LPS stimulation of inflammatory cytokines in N9 cells. Cells were incubated with PEA 100 µM for 1 h and then LPS 3 µg/mL was added for 6 h. mRNA levels of (**A**) tumor necrosis factor alpha (TNF-α), (**B**) interleukin-6 (IL-6), (**C**) monocyte chemoattractant protein-1 (MCP-1), (**D**) interleukin-1 beta (IL-1β), (**E**) interleukin-10 (IL-10) and (**F**) toll-like receptor 4 (TLR4) were examined by real-time PCR. β-actin mRNA was used for data normalization. Data are shown as the mean ± S.E.M. of measurements obtained in three independent experiments (*n* = 18). (**G**) Release of TNF-α into the culture medium was quantified by using a specific enzyme-linked immunosorbent assay (ELISA). Data are shown as the mean ± S.E.M. of measurements obtained in three independent experiments (*n* = 3). (**H**) IL-1β protein expression was measured by Western blotting. To ensure equal loading, blots were also probed with anti-GAPDH. Data are shown as the mean ± S.E.M. of measurements obtained in three independent experiments (*n* = 3). Differences between groups were tested for significance by applying the ANOVA test and Tukey post hoc test. ** *p* < 0.01, *** *p* < 0.001, **** *p* < 0.0001.

To further characterize the role of PEA on microglia activation and polarization, inflammatory cytokine levels were measured in N9 cells after LPS incubation. LPS stimulated an increase of mRNA levels for TNF-α, interleukin-6 (IL-6), and IL-1β (Figure 2A,B,D, respectively), as well as those for monocyte chemoattractant protein-1 (MCP-1; Figure 2C), which are all markers of the M1 phenotype. In addition, LPS induced an increase also in mRNA levels for interleukin-10 (IL-10) (Figure 2E), which is a marker of the M2b microglia sub-phenotype. The pre-treatment with PEA 100 µM significantly antagonized LPS stimulation of mRNA levels for pro-inflammatory cytokines, as well as the increase in IL-10. Moreover, PEA effectively reduced TNF-α release in the culture medium (Figure 2G) and the cellular content of pro-IL-1β protein (Figure 2H). These findings suggest that PEA pre-treatment largely counteracts microglia activation induced by LPS, preventing the classical M1 microglia polarization. It is well known that LPS polarizes microglia to a mixed M1/M2b phenotype through the toll-like receptor (TLR) signaling. Thus, we measured the mRNA levels of TLR4 in N9 cells. The incubation with LPS induced a significant reduction of TLR4 mRNA levels, and this effect was not inhibited by the pre-incubation for 1 h with PEA (Figure 2F). The ability of LPS to downregulate TLR4 has been already demonstrated in immortalized microglia cells [8], in primary culture [9], and in macrophages [10], and it has been suggested that this may be a mechanism to prevent over stimulation of the innate response.

### 2.2. PEA Pre-Treatment Prevents Microglia De-Ramification Induced by LPS Treatment

**Figure 3:**
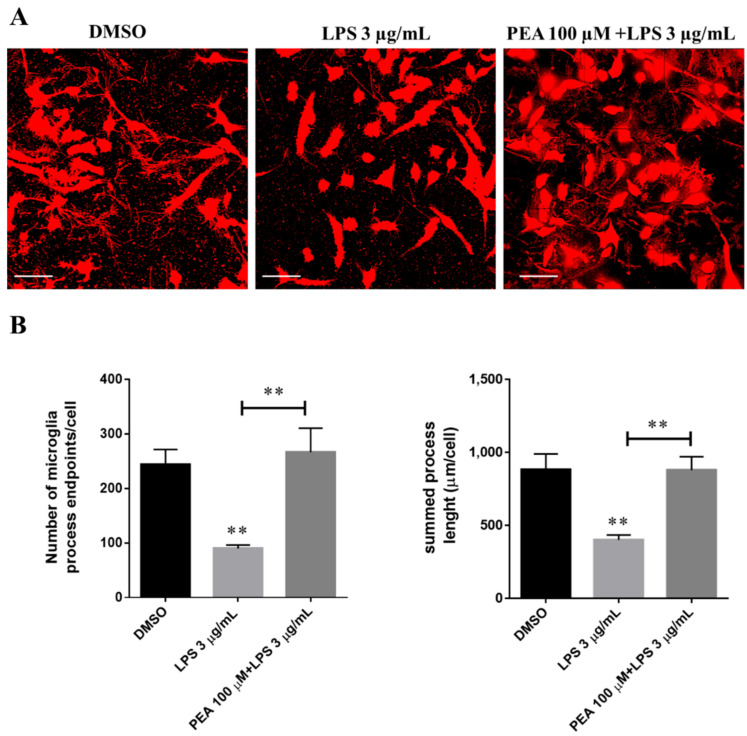
Effect of PEA on microglia ramification in LPS-stimulated N9 cells. N9 cells were seeded on porcine gelatin pre-treated coverslips and then were incubated with 100 µM PEA at 1 h before treatment with 3 µg/mL LPS for 6 h. (**A**) Cells were stained with the membrane dye DiI and fixed. Fluorescence images were acquired by a 63× magnification on Leica TCSSP2 laser scanning confocal microscope. Scale bar: 50 µm. (**B**) Histograms relative to the quantification of process endpoints/cells and process length/cells. Data are shown as the mean ± S.E.M. of different images analyzed (*n* = 5, total number of cells present in each image *n* = 30, total number of cells analyzed for each group *n* = 150). Differences between groups were tested for significance by applying the ANOVA test and Tukey post hoc test. ** *p* < 0.01.

In order to further evaluate microglia polarization, N9 cell morphologies were analyzed after LPS exposure. Microglia morphology was visualized by a confocal microscope. As shown in Figure 3A, under control condition, microglia exhibited the typical ramified morphology of resting microglia (M0 stage) with numerous long branches and multiple filopodia [11]. As expected, LPS administration changed microglia morphology from the typical branched and ramified morphology to amoeboid with loss of most branches (M1 stage). The morphological changes in microglia reflect profound functional changes in these cells, because it is known that the release of cytokines and other signaling factors into the surrounding tissue is enhanced when microglia acquire amoeboid morphology [12], in agreement with our previous results on cytokine expression. PEA pre-treatment prevents LPS-induced microglia shift into M1 state, keeping cells in ramified morphology. On this basis, microglia ramified morphology changes were measured by quantifying the number of microglia endpoints and process length per cell. As shown in Figure 3B, microglia endpoints and process length significantly decreased with LPS treatment compared to control, while these effects were no present in cells pre-treated with 100 µM PEA for 1 h. Therefore, skeleton analysis of microglia morphologies revealed that microglia became de-ramified (fewer and shorter processes per cell) in LPS treatment while PEA pre-treatment counteracted this situation maintaining cells in ramified form.

**Figure 4:**
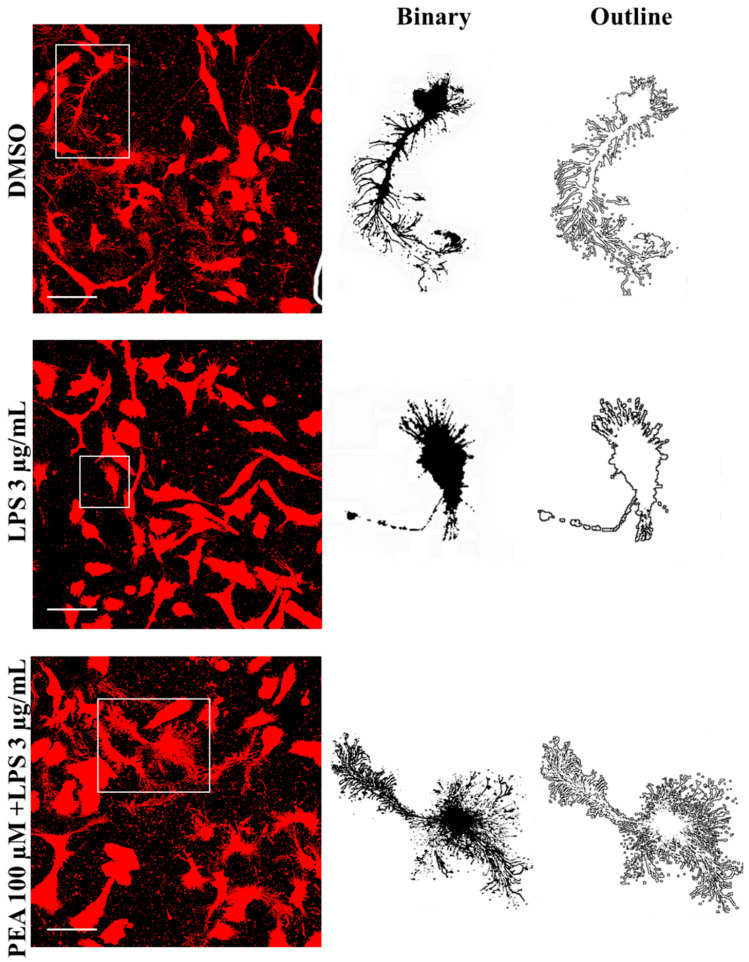
Effect of PEA on microglia morphology in LPS-stimulated N9 cells. N9 cells were seeded on porcine gelatin pre-treated coverslips and then were incubated with 100 µM PEA at 1 h before treatment with 3 µg/mL LPS for 6 h. Cells were stained with the membrane dye DiI and fixed. Fluorescence images were acquired by a 63× magnification on Leica TCSSP2 laser scanning confocal microscope. On the right, examples of cell outlines. Scale bar: 50 µm.

Furthermore, to better analyze microglia morphological changes, FracLac analysis was performed. Examples of microglia (made binary and outlined) in each treatment are shown in Figure 4.

**Figure 5:**
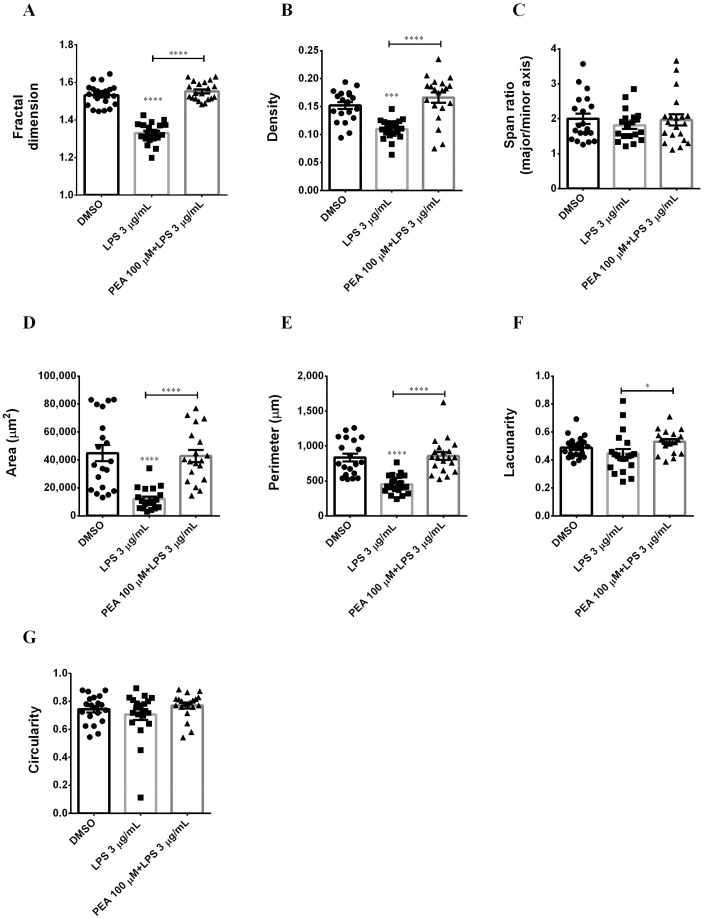
Effect of PEA on microglia complexity, elongation, and size in LPS-stimulated N9 cells. Summary data and statistical analysis of fractal dimension (**A**), density (**B**), span ratio (**C**), area (**D**), perimeter (**E**), lacunarity (**F**), and circularity (**G**). Total number of cells analyzed for each condition *n* = 20. Data are shown as the mean ± S.E.M. Differences between groups were tested for significance by applying the ANOVA test and Tukey post hoc test. * *p* < 0.05, *** *p* < 0.001, **** *p* < 0.0001.

Application of FracLac for ImageJ to microglia outlines resulted in fractal dimensions that ranged from 1.33 and 1.532–1.542 (available range is 1–2), with the lowest value occurring in LPS treatment and the highest in control and PEA pre-treatment. Indeed, microglia complexity significantly decreased with LPS administration, while PEA pre-incubation counteracted it (Figure 5A). Fractal dimension scores suggest that PEA pre-treatment is able to maintain complexity of microglia shape, which characterizes control cells. Using FracLac for ImageJ, additional measures of microglia morphology related to cell shape were investigated: span ratio, density, cell area, perimeter, lacunarity, and circularity. Span ratio is a measure of microglia elongation. Therefore, this parameter, together with cell circularity, is relevant to define microglia shape (rod or circle). As shown in Figure 5C,G, span ratio and cell circularity were unchanged among different groups. Density, cell area, and perimeter are used to report microglia size. LPS administration decreased density, cell area, and perimeter compared to control, while they were significantly preserved in cells pre-treated with 100 µM PEA for 1 h (Figure 5B,D,E). Lacunarity (Λ) is a measure of microglia shape changing; since Λ assesses heterogeneity or translational and rotational invariance in an image, lower Λ values imply homogeneity [13]. As shown in Figure 5F, PEA pre-treatment significantly increased lacunarity compared to LPS administration.

**Figure 6:**
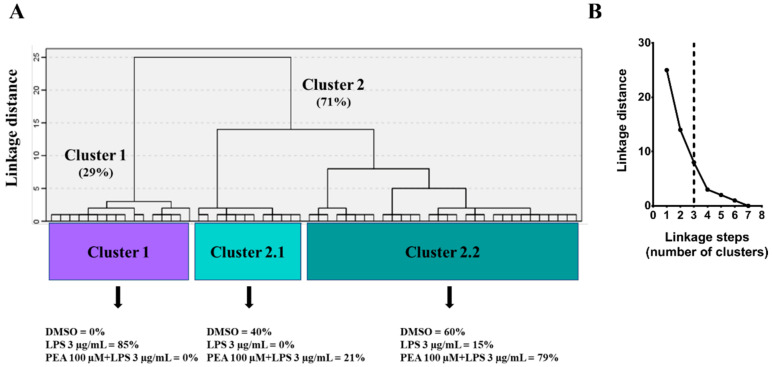
Classification of microglia according to FracLac parameters. (**A**) Hierarchical cluster analysis (HCA) of microglial cells, treated as described above, based on parameters calculated by FracLac analysis. Dendrogram for 59 cells, where the abscissa represents individual microglia, and the ordinate corresponds to the linkage distance measured by Euclidean distance. As shown, data were classified as two main clusters (Cluster 1 and 2). Cell morphology of LPS-treated microglia fit into Cluster 1; on the contrary, both not treated cells and cells pre-treated with PEA belong to the second Cluster (Clusters 2.1 and 2.2) (**B**) Three was identified as an appropriate number of clusters according to the linkage distance (Euclidean distance) vs linkage steps (number of clusters) analysis performed following Thorndike’s procedure, as indicated by the vertical dashed line.

To properly categorize the different morphotypes of microglia present in this experimental model and correlate them with their activation state, hierarchical cluster analysis (HCA) was performed (Figure 6A). For this mathematical approach to classify microglia in different groups, parameters calculated by FracLac analysis were used. Based on these measures, and following Thorndike’s procedure [14], microglia were classified in three clusters or morphotypes (Figure 6B). Especially, as shown in Figure 6A, there were two big clusters, Cluster 1 and 2, with Cluster 2 further ramified in two sub-groups, 2.1 and 2.2. Cluster 1 included cells from LPS-treatment, while Cluster 2 included a mixture of cells from both control and PEA pre-treatment. Notably, whereas control cells were equally distributed between Cluster 2.1 and Cluster 2.2, the greatest part of cells derived from PEA pre-incubation were grouped in Cluster 2.2.

**Figure 7:**
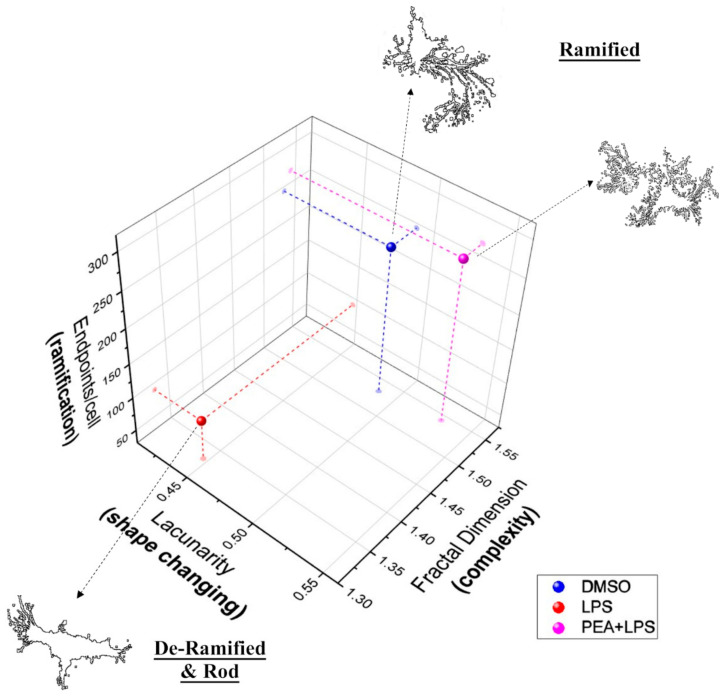
Effect of PEA on different microglia morphologies in LPS-stimulated N9 cells. Fractal dimension (D_B_), endpoints/cell, and lacunarity data were analyzed by Pearson’s correlation and then averages for each group were used for visualization. The figure summarizes the relationship between all three variables. Fractal dimension (D_B_) is directly related to endpoints/cell (*r* = 0.9994, *p* = 0.0108) and lacunarity (Λ) (*r* = 0.2637, *p* = 0.06954); lacunarity (Λ) is not significantly correlated with endpoints/cells (*r* = 0.9161, *p* = 0.1313). Two groupings emerge: ramified and de-ramified and rod.

To better understand microglia morphology and its changes, which reflect profound functional modifications, endpoints/cell, fractal dimension, and lacunarity score from different treatments were plotted on a 3D graph; data were averaged for each group. These three measures were chosen as single variables to represent cell ramification, complexity, and shape changing, respectively. Including all variables would be redundant. Plotting these three parameters on a scatter plot (Figure 7) allowed us to categorize PEA pre-treatment in the same group of control (ramified group) as opposed to LPS treatment (de-ramified and rod group). Even though PEA and control group were near in the plot, PEA group displayed a higher lacunarity. Lacunarity defines the polarization of the cells, which is where their prolongations are oriented toward a specific point, and this characteristic is typical of the M2a phenotype.

### 2.3. PEA Inhibits ATP-Induced Intracellular Ca^2+^ Increase in N9 and Primary Microglial Cells

**Figure 8:**
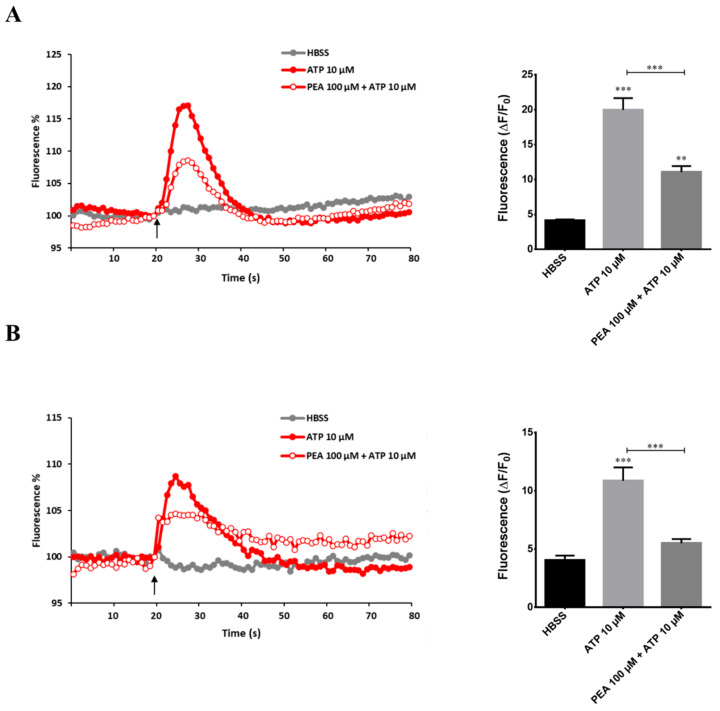
Effect of PEA on ATP-mediated intracellular Ca^2+^ increase in primary microglia and N9 cells. Cells were loaded with FLUO-4 NW and fluorescence emission was measured at 535 nm every 0.5 s for the 20 s preceding and the 60 s following the injection of the stimuli. (left) ATP was injected in (**A**) N9 cells and in (**B**) primary microglia at the time indicated by the arrow. Results are the means of measurements obtained in at least six different wells for each experiment. All experiments were repeated at least three times. One representative experiment is shown (right). Graphs show intracellular Ca^2+^ increase (expressed as fluorescence intensity) in (**A**) N9 cells and (**B**) primary microglia stimulated with ATP. Data are shown as the mean ± S.E.M. of measurements obtained in three independent experiments (*n* = 18). Differences between groups were tested for significance by applying the ANOVA test and Tukey post hoc test. ** *p* < 0.01, *** *p* < 0.001.

Microglia function strongly relies on intracellular calcium signaling. Unable to observe substantial increase in calcium following LPS application, we studied the calcium elevation induced by ATP. In particular, microglia expressed receptors for ATP that regulate microglial motility. After local damage, the release of ATP induced microgliosis and activated microglial cell migration to the site of injury, proliferation, and phagocytosis of cells and cellular compartments [15]. To investigate if PEA could affect the calcium signaling in microglia, we evaluated intracellular Ca^2+^ mobilization in N9 cells. Stimulation of cells with 10 µM ATP caused a transient increase of intracellular Ca^2+^ that was significantly reduced (approximately 45%) by the pre-treatment with 100 µM PEA (Figure 8A). Increase in cytoplasmic Ca^2+^ ion concentration creates that phenomenon called intercellular Ca^2+^ wave (ICW), which is the main communication approach by which glial cells interact and coordinate with each other to execute immune defense [16,17]. In order to obtain further insight about the role of PEA in counteracting intracellular Ca^2+^ increase induced by ATP, we repeated the experiment in primary microglial cells. According to what we observed for N9 cells, pre-treatment of primary microglia with 100 µM PEA inhibited (49%) the rise in intracellular Ca^2+^ concentration induced by ATP (Figure 8B). These data suggest that PEA is able to prevent generalized Ca^2+^ transients that in microglia are a signal of damage.

### 2.4. PEA Prevents LPS-Induced Hyperexcitability in Primary Cortical Cultures

**Figure 9:**
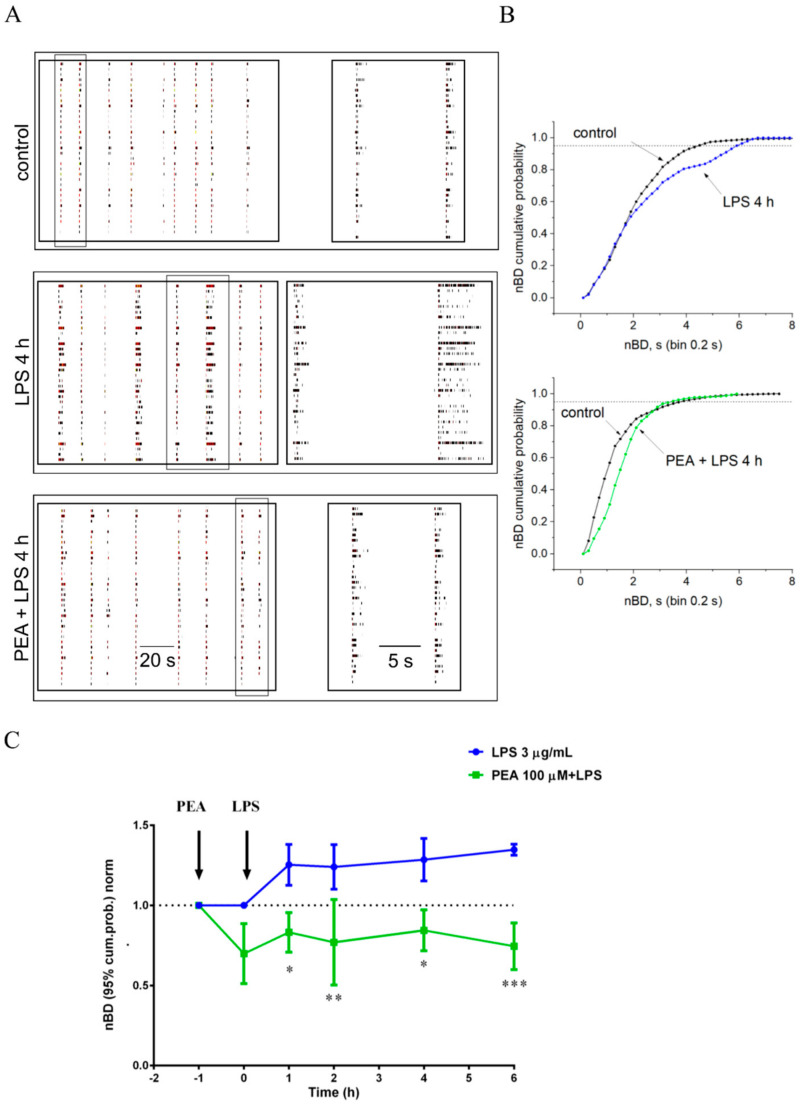
Protective effect of PEA on LPS-induced inflammation and hyperexcitability of cortical network. (**A**) Representative example of raster plot of patterns of spontaneous burst activity in control condition after 4 h of 3 μg/mL LPS administration and during 100 μM PEA + LPS. PEA was administered 1 h before LPS. In each condition, a couple of bursts were boxed and shown in a new window with a time scale enlarged, on the right of panel A. Each vertical line is a timestamp representing a single spike, and the global network burst duration (BD) is computed considering all neurons (distributed on the row). (**B**) Plots of superimposed BD cumulative probability histograms in control (black line), after 4 h of LPS administration (upper panel) and LPS with PEA pre-administered (lower panel). The data are obtained, computing all the BDs in temporal windows of 30 min, then, the cumulative probabilities of the time lapse of interest were normalized to the maximum value. Number of analyzed bursts in represented experimental steps were 110 in control, 103 in LPS, and 108 in PEA + LPS. On cumulative distribution, the statistical significance, obtained by Kruskal–Wallis non parametric test, was *p* < 0.01 for LPS vs control and *p* > 0.05 for PEA + LPS vs control. The value of cumulative distribution of the burst durations (cumBD) at 95% of probability (dot line) in the upper panel changed from 4.4 s, in control, to 6 s, in LPS; in the lower panel the same value is 3.6 s in control and 3.55 s in PEA + LPS condition. (**C**) Time plot of cumBD data at 95% of probabilities; each point in the indicated time lapse represents averaged data normalized at the control (dot line). Significant differences for PEA + LPS (green line) vs LPS (blue line) were determined at all experimental steps by Holm–Sidak method. 0.832 ± 0.123 and 1.253 ± 0.128, 0.769 ± 0.267 and 1.240 ± 0.139, 0.844 ± 0.127 and 1.285 ± 0.132, 0.745 ± 0.146 and 1.347 ± 0.034 were, respectively, the values for PEA + LPS and LPS at 1, 2, 4, and 6 h (*n* = 3 independent experiments for each condition). No significant effect was observed in PEA versus control. Data are expressed as mean ± S.E.M. * *p* < 0.05, ** *p* < 0.01, *** *p* < 0.001.

LPS treatment of cultures of neurons, astrocytes, and microglia causes atypical seizure-like activity in the neuronal network, which we have characterized in detail in our previous studies [18,19]. To investigate if PEA could counteract the hyperexcitability induced by LPS, we pre-incubated primary cortical cultures with 100 µM PEA 1 h before administering 3 µg/mL LPS. The electrical activity of the network, recorded by the MEA system, is shown in the raster plots in Figure 9A for a representative culture in control, during LPS administration, and during PEA pre-administered to LPS. In the raster plot, each line corresponds to one cell, and the small vertical ticks represent the spikes. PEA abolished the atypical hyperactivity induced by LPS and, in some cultures, it even showed a trend to reduce the network activity compared to control condition. In order to more precisely characterize the effect of PEA, we evaluated the cumulative distribution of the burst durations (cumBD) of the network, which in our previous papers was considered as the principal variable for describing the atypical seizure-like events induced by LPS. The typical effect of LPS on cumBD was a right-shift of the curve with respect to control (Figure 9B, upper panel). From the figure it is evident that, in control, 95% of the bursts had BD < 4 s, whereas in LPS, only ~80% of the bursts had BD < 4 s and the rest of the bursts had longer BDs. Pre-incubation with PEA increased to 100% the probability of having BD < 4 s (Figure 9B, lower panel), showing a preventive effect of PEA on the LPS treatment. In order to compare different experiments, the value of cumBD at 95%, which represented the duration of 95% of the bursts (cumBD95), was extracted for LPS and PEA + LPS from each experiment and was normalized on cumBD95 of its respective control; thus, data from *n* = 3 independent experiments were averaged. Data at different time points (0, 1, 2, 4, and 6 h from LPS administration) were represented in Figure 9C in order to show the time course of LPS administration without and with PEA pre-incubation. PEA counteracted LPS-induced increase in cumBD95 at all the investigated time points (*p* < 0.05 at 1 and 4 h, *p* < 0.01 at 2 h, *p* < 0.001 at 6 h), suggesting a long-lasting preventive action of PEA on network hyperexcitability caused by LPS. The reduction of network activity shown by PEA administration versus control was not significant (*p* > 0.05, multiple *t*-test corrected for multiple comparisons by using the Holm–Sidak method).

### 2.5. PEA Counteracts LPS-Induced TNF-α Release in Primary Cortical Cultures

**Figure 10:**
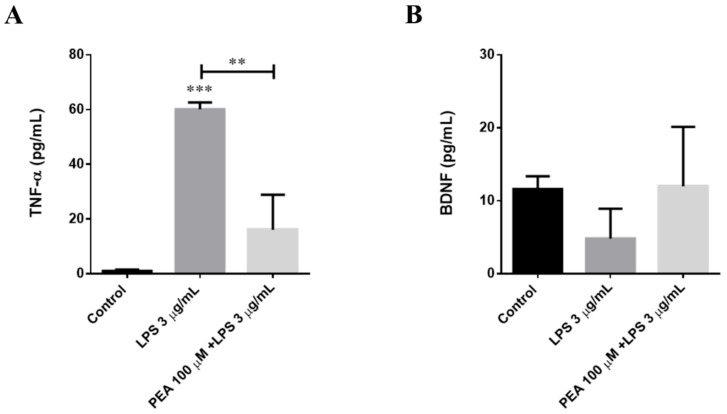
Release of TNF-α (**A**) and BDNF (**B**) at 6 h after LPS application. The mouse primary cortical cultures were incubated with 100 µM PEA at 1 h before treatment with 3 µg/mL LPS. The amount of TNF-α and brain derived neurotrophic factor (BDNF) released into the culture medium was quantified after 6 h from LPS treatment. Data are shown as the mean ± S.E.M. of measurements obtained in three independent experiments (*n* = 3). Differences between groups were tested for significance by applying the ANOVA test and Tukey post hoc test. ** *p* < 0.01, *** *p* < 0.001.

As described above, TNF-α is an inflammatory cytokine marker of the M1 microglia polarization state. To verify whether PEA pre-treatment is able to counteract the microglial-release of TNF-α induced by LPS treatment in primary cortical neuron/astrocyte/microglia cultures, we performed an ELISA. The amount of this cytokine in the presence of PEA was, at 6 h after LPS treatment, significantly smaller than that found when PEA was absent (Figure 10A). Thus, to assess if PEA pre-treatment activates anti-inflammatory processes in primary cortical cultures, we measured the level of brain derived neurotrophic factor (BDNF). As shown in Figure 10B, the amount of this neurotrophin did not increase compared to control. These findings confirm what we previously observed in N9 microglia cells, namely, that PEA pre-treatment can counteract inflammation induced by LPS treatment.

### 2.6. PEA Counteracts LPS-Induced Nf-κB Activation in Juman PMA-THP-1 X-Blue Cells and This Effect Is Not Mediated by Interaction with TLR4

**Figure 11:**
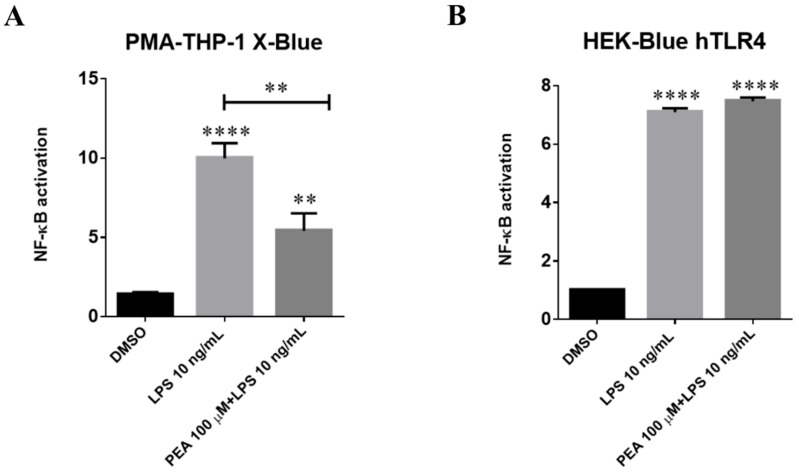
Inhibition of nuclear factor kappa-light-chain-enhancer of activated B cells (NF-κB) activation in LPS-stimulated PMA-THP-1 X-Blue cells by PEA. PMA-THP-1 X-Blue (**A**) and HEK-Blue hTLR4 (**B**) cells were incubated with 100 µM PEA at 1 h before treatment with 10 ng/mL LPS. The amount of secreted embryonic alkaline phosphatase (SEAP) released into the culture medium was quantified after 6 h as a measure of NF-κB activation. Data are presented as mean ± S.E.M. (*n* = 3 independent experiments) normalized on the control sample. Differences between groups were tested for significance by applying the ANOVA test and Tukey post hoc test. ** *p* < 0.01, **** *p* < 0.0001.

LPS is a ligand for TLR4. When LPS is bound together with TLR4, a cascade of signaling pathways is triggered, which can activate the expression of pro-inflammatory cytokines, including interleukin (IL)-1, IL-6, and TNF-α [20]. Although structurally different, TNF-α, IL-1, and TLR receptors use similar signal transduction mechanisms that include activation of IkB kinase (IKK) and nuclear factor kappa-light-chain-enhancer of activated B cells (NF-κB) [21]. In recent years, it has become clear that there are at least two separate pathways for NF-κB activation. The “canonical” pathway is triggered by microbial products and proinflammatory cytokines such as TNF-α and IL-1, usually leading to activation of RelA- or cRel-containing complexes [22]. Moreover, preclinical studies showing the therapeutic effect of synthetic small molecules acting as TLR4 antagonists, both in vitro and in vivo, confirmed its central role in the regulation of inflammation [23]. In order to obtain further insight about the role of TLR4/NF-κB axis in the previously observed PEA-induced inhibition of pro-inflammatory cytokines, we investigated NF-κB activation triggered by TLR4 stimulation in human macrophages PMA-THP-1 X-Blue cells. Cells were pre-incubated with 100 µM PEA 1 h before administering 10 ng/mL LPS. As shown in Figure 11A, secreted embryonic alkaline phosphatase (SEAP) expression significantly decreased (40%) in human macrophages upon PEA pre-treatment as compared to LPS, accordingly to previous report [24]. PEA is able to inhibit activation of NF-κB even if PEA is administered after LPS in THP-1 derived macrophages. Taken together, these findings could suggest a possible involvement of the TLR4/NF-κB axis in the PEA mechanism of action in human macrophages.

To confirm this hypothesis, we investigated NF-κB activation in HEK-Blue hTLR4 cells. HEK-Blue hTLR4 cells served as a tool to investigate whether PEA acts involving TLR4 directly. Pre-treatment with PEA did not affect NF-κB activation induced by LPS (Figure 11B). These data indicated that PEA is able to inhibit NF-κB activation triggered by LPS administration in human macrophages, but this effect is not mediated by interaction with TLR4.

### 2.7. CB2 Receptor Is Partially Involved in PEA-Induced Effects on Microglia

**Figure 12:**
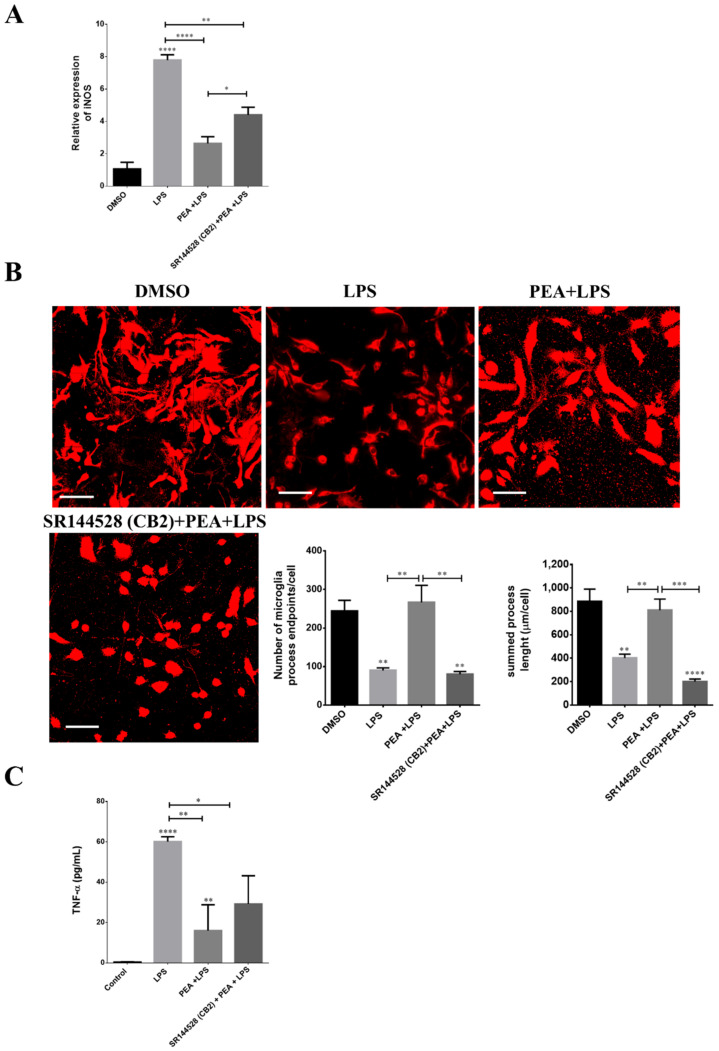
Involvement of cannabinoid type 2 receptor (CB2R) in PEA-neuroprotective effects. N9 cells and mouse primary cortical cultures were incubated with 100 nM SR144528 at 1 h before 100 µM PEA pre-treatment. After 1 h, they were then exposed to 3 µg/mL LPS. (**A**) Histograms relative to the quantification of Western blot bands of iNOS in N9 cells, after DMSO, LPS, PEA + LPS and SR144328 + PEA + LPS treatments. Data are shown as the mean ± S.E.M. (*n* = 3 independent experiments) (**B**) N9 cells were seeded on porcine gelatin pre-treated coverslips and treated as described above. Cells were stained with the membrane dye DiI and fixed. On the left, fluorescence images acquired by a 63× magnification on Leica TCSSP2 laser scanning confocal microscope. Scale bar: 50 µm. On the right, histograms relative to the quantification of process endpoints/cells and process length/cells. Data are shown as the mean ± S.E.M. of different images analyzed (*n* = 5, total number of cells present in each image *n* = 30, total number of cells analyzed for each group *n* = 150). (**C**) The amount of TNF-α released into the culture medium in mouse primary cortical cultures. Cells were treated as described above, and the amount of this cytokine was quantified at 6 h from LPS application. Data are shown as the mean ± S.E.M. of measurements obtained in three independent experiments (*n* = 3). Differences between groups were tested for significance by applying the ANOVA test and Tukey post hoc test. * *p* < 0.05, ** *p* < 0.01, *** *p* < 0.001, **** *p* < 0.001.

Microglia express both the cannabinoid type 1 (CB1R) and type 2 (CB2R) receptors; however, CB2R is more abundantly expressed in microglial cells [25], and its expression is further increased during activation in vitro and in disease animal models [26]. Therefore, it is expected that CB2R plays a crucial role in the anti-inflammatory microglial response. In fact, upregulation of the alternative M2 markers by CB2R activation in microglial cells has been reported [27]. To investigate the role of CB2R in PEA-neuroprotective effects, a selective CB2R inverse agonist SR144528 was used. The expression of iNOS was measured as the marker of microglial activation by Western blot analysis. As shown in Figure 12A, pre-administration of SR144528 100 nM significantly increased iNOS expression compared to PEA treatment. The ability of SR144528 to partially antagonize PEA effects could be due to a possible action of PEA on CB2R. As described above, the morphological changes in microglia reflect profound functional modifications. On this basis, microglia ramified morphology changes were quantified. The benefits induced by PEA pre-treatment, which counteracted the de-ramified form induced by LPS, were completely reversed in presence of SR144528 (Figure 12B). Moreover, in primary cortical neuron/astrocyte/microglia cultures, pre-incubation with SR144528 100 nM significantly increased TNF-α release compared to control, partially antagonizing PEA preventive effects (Figure 12C). Taken together, these findings suggest a partial involvement of CB2R in the PEA mechanism of action.

## 3. Discussion

In this study, characterized the effects of PEA in several in vitro models of neuroinflammation induced by LPS and ATP. Our results demonstrated that: (i) PEA induced a switch from M1 to M2 phenotype in N9 microglia stimulated with LPS; (ii) PEA blunted the increase of intracellular calcium stimulated by ATP in N9 and primary microglia; (iii) PEA antagonized the hyperexcitability in cultures of primary cortical neurons, astrocytes and microglia induced by LPS; and (iv) PEA inhibited NF-κB activation by modulating the activation of CB2R signaling but not through interactions with TLR4.

PEA is an endogenous lipid messenger which displays key properties in many biological processes, showing not only anti-inflammatory activities but also analgesic and neuroprotective ones [28]. For this reason, PEA is potentially effective in a wide range of therapeutic areas, as demonstrated by many preclinical and clinical studies that highlighted its efficacy in neurodegenerative diseases [29], chronic pain conditions [30], and epilepsy [4]. The efficacy and the tolerability of PEA explain why it has been marketed in different countries as a nutraceutical food supplement since 2008. Different molecular mechanisms have been proposed so far to explain the biological effects of PEA, including direct action on proliferator-activated receptor-α (PPAR-α) [31] and many indirect cannabinoid receptor-mediated actions through the so-called entourage effect [32]. In spite of this multiple mechanism of action, the effect of PEA on cell types involved in inflammatory responses has not been yet fully characterized. In the present study, we focused on the mechanisms by which PEA modulates the release of pro- and anti-inflammatory mediators from microglia and its capability to shift microglia from M1 to M2 polarization.

A growing body of evidence suggests an important role for neuroinflammation in the pathogenesis of several neurological diseases, including, among others, amyotrophic lateral sclerosis, Alzheimer’s, and Parkinson’s diseases [33]. It is generally acknowledged that acute neuroinflammation plays a protective role in the CNS by activating microglial phagocytosis of harmful agents, whereas the chronicization of neuroinflammation assumes a detrimental role because of the excessive release of inflammatory cytokines and cytotoxic factors [34]. Microglia are derived from myeloid cells in the periphery and comprise approximately 12% of cells in the brain, where they typically exist in a resting state characterized by ramified morphology [35]. Microglia are highly plastic cells, and classification of activated microglia into the three classical phenotypes [i.e., (i) resting M0; (ii) pro-inflammatory M1; and (iii) immunosuppressive M2] is the most commonly used approach to represent a simplified model of polar extremes of inflammatory response [36]. A switch from M1 to M2 microglia is thought to occur during natural resolution of inflammation, and M2 microglia are often described as having anti-inflammatory or reparative functions [37]. In the present study, we used LPS to stimulate pro-inflammatory responses in the N9 murine microglia cells, which were previously demonstrated to be a suitable model for pharmacological and toxicological studies on microglia [38,39]. Our results in N9 microglia demonstrated that LPS stimulated an increase in the expression of pro-inflammatory mediators, including iNOS, IL-1β, TNF-α, IL-6 and MCP-1, and inhibited the expression of Arg1. Interestingly, LPS stimulation inhibited the expression of TLR4, which exists as a complex with the co-receptor myeloid differentiation protein-2 (MD-2) [40]. The binding of LPS to TLR4-MD-2 complexes activated downstream mediator pathways, including NF-κB. This ability of LPS to downregulate TLR4 may be a negative feedback mechanism to prevent over stimulation of the innate response [8,9,10]. Also, at the morphological level, LPS induced a shift of N9 microglia from the M0 to M1 amoeboid shape. The morphological changes in microglia morphology are indicative of primary functional modification since it is known that the release of cytokines and other signaling factors into the surrounding tissue is enhanced when microglia acquire amoeboid morphology [12]. Interestingly, PEA effectively antagonized the effects of LPS on pro-inflammatory mediators and IL-10 expression and significantly stimulated the anti-inflammatory Arg-1 expression. These effects were also reflected in the ability of PEA to induce LPS-stimulated amoeboid microglia to switch toward the M2 shape. Additionally, fractal dimension analysis suggested that PEA was capable to preserve the complexity of microglia shape, similar to control, and also in cells stimulated with LPS. However, N9 treated with PEA displayed a higher lacunarity, a characteristic that defines cell polarization, indicating that their prolongations were oriented toward a specific point, and this characteristic is typical of M2a phenotype. Interestingly, PEA was unable to restore TLR4 expression reduced by LPS, suggesting that its intracellular mechanism of action is at least in part independent from that of LPS.

Microglia function strongly relies on intracellular calcium signaling. In fact, release of ATP from damaged cells stimulates microglial cell migration to the site of injury, proliferation, and phagocytosis of cells [15]. Extracellular ATP is an important signal for the intercellular Ca^2+^ wave (ICWs) [41,42,43], which is the primary mechanism by which microglia [16,17], astrocytes [44], and neurons [45] communicate to maintain homeostasis of the CNS. In N9 cells, stimulation with ATP caused a significant transient increase of intracellular Ca^2+^ that lasted for about 30 s. PEA pre-treatment significantly inhibited the increase of intracellular Ca^2+^ stimulated by ATP. Almost superimposable effects were obtained also in murine primary microglia, demonstrating that: (i) PEA can modulate the intracellular activation initiated by binding of ATP to purinergic receptors; and (ii) N9 cells are a reliable experimental model to study the effects of pharmacological treatments on microglia.

In cultures of neurons, astrocytes, and microglia, we evaluated the cumulative distribution of the burst durations (cumBD) of the network, which can be assumed as the principal variable for describing the atypical seizure-like events [18,19]. In the control group, about 95% of the bursts had BD < 4 s, but in the group stimulated with LPS, only ~80% of the bursts had BD < 4 s. This effect could be mediated, at least in part, by LPS stimulation of TNF-α release in the culture medium. TNF-α was previously shown to control basal synaptic functions [46] as well as plasticity [47,48]. In this setting, PEA effectively increased to 100% the probability of having BD < 4 s in cultures stimulated with LPS, and significantly inhibited the release of TNF-α in the culture medium. These data demonstrated that PEA has important modulatory activity and can prevent network hyperexcitability caused by LPS treatment.

More insights into the PEA mechanism of action were obtained using both the PMA-THP-1 X-Blue cell and HEK-Blue hTLR4 models. These cells have been modified for measuring NF-κB activation in terms of SEAP release in the culture medium. Our results showed that LPS significantly increased NF-κB activation in PMA-THP-1 X-Blue cells, and this effect could be significantly inhibited by PEA pre-incubation. However, in HEK-Blue hTLR4, PEA was unable to inhibit LPS stimulation of NF-κB activation, suggesting that its effects are not mediated by a direct effect on TLR4 receptors. Previous evidence suggested a role of CB2 receptor in the mechanism of action of PEA. To investigate the role of CB2R in PEA-anti-inflammatory effects, we used SR144528, a selective CB2R inverse agonist. SR144528 significantly reduced the ability of PEA to inhibit LPS stimulation of iNOS expression in N9 cells. Similarly, SR144528 was able to antagonize PEA capability to shift the morphology of N9 cells treated with LPS from the M1 to M2 shape. It has been reported that PEA does not directly bind CB1R or CB2R, and our results are in agreement with those suggesting that PEA anti-inflammatory effects could result by its ability to stimulate a yet unknown CB2-like receptor [49].

In conclusion, the present study provides evidence that PEA is an effective inhibitor of the pro-inflammatory effects of LPS in microglia. PEA can also modulate microglia activation induced by ATP stimulation. We also demonstrated that PEA can effectively counteract microglial morphological changes induced by LPS and hyperactivation of neurons–microglia–astrocyte networks. PEA effects are likely mediated by its interaction with a still unknown CB2-like receptor. These findings suggest that PEA could be a useful drug to prevent the consequences of chronic neuroinflammation in neurodegenerative disorders.

## 4. Materials and Methods

### 4.1. Cell Cultures

The murine microglial N9 cells were cultured in Iscove Modified Dulbecco’s Medium (IMDM, Sigma-Aldrich, St. Louis, MO, USA) supplemented with 5% heat-inactivated fetal bovine serum (FBS), 100 U/mL penicillin, 100 μg/mL streptomycin, 2 mM l-glutamine (all Euroclone, Pero, Italy), and Mycozap Prophylactic (Lonza, Walkersville, MD, USA) under standard cell culture conditions (37 °C, 5% CO_2_).

Primary microglial cells were isolated from P2 neonatal rats as previously described [50]. Briefly, isolated hippocampi and cortices were triturated and suspended in complete glial medium, composed of Minimum Essential Medium (MEM, Sigma-Aldrich, St. Louis, MO, USA), 20% FBS (Euroclone, Pero, Italy), 33 mM glucose (Sigma-Aldrich, St. Louis, MO, USA), 2 mM ultra-glutamine (Lonza, Walkersville, MD, USA), 100 U/mL penicillin, and 100 μg/mL streptomycin (Euroclone, Pero, Italy). Cells were then seeded in 0.02 mg/mL poly-d-lysine pre-coated flasks and cultured under standard cell culture conditions (37 °C, 5% CO_2_) for 15 days. After this period, microglial cells were isolated from mixed glial cultures by shaking flasks and then seeded onto 0.05 mg/mL poly-ornithinated plates at the desired concentration.

THP-1 X-Blue cells (InvivoGen, San Diego, CA, USA), derived from a human peripheral blood monocyte cell line by stable integration of an NF-κB-inducible SEAP reporter construct, were obtained. Cells were maintained in RPMI 1640 Medium without l-Glutamine with Phenol Red (Euroclone, Pero, Italy), supplemented with 10% heat-inactivated FBS, 2 mM l-Glutamine, and 100 U/mL Penicillin/Streptomycin (all Euroclone, Pero, Italy). Before treatments, cells were seeded into a 96-well plate and differentiated into macrophages by 72 h incubation with 100 ng/mL phorbol 12-myristate 13-acetate (PMA; Enzo Life Sciences, New York, NY, USA), followed by 24 h incubation in RPMI medium without PMA.

HEK-Blue hTLR4 cells (InvivoGen, San Diego, CA, USA) were obtained by co-transfection of the human TLR4, MD-2 and CD14 co-receptor genes, and an inducible SEAP reporter gene into HEK293 cells. Cells were maintained in DMEM medium without l-Glutamine with Phenol Red (Euroclone, Pero, Italy), supplemented with 10% heat-inactivated FBS, 2 mM l-Glutamine, 100 U/mL Penicillin/Streptomycin (all Euroclone, Pero, Italy), and HEK-Blue Selection (InvivoGen, San Diego, CA, USA), a solution that combines several selective antibiotics to maintain selection pressure.

### 4.2. Chemicals

PEA was purchased from Cayman Chemical (Ann Arbor, MI, USA). It was dissolved in dimethyl sulfoxide (DMSO) at a concentration of 67 mM or 100 mM and then serially diluted in culture medium immediately prior to experiments. Adenosine 5′-triphosphate (ATP) and lipopolysaccharides (LPS; *Escherichia coli* O55:B5) were obtained from Sigma-Aldrich (St. Louis, MO, USA). SR144528 was purchased from Tocris (Bristol, UK).

### 4.3. Real-Time PCR Analysis

N9 cells were plated in 24-well culture plates at a density of 80 × 10^3^ cells/well and incubated at 37 °C for 48 h. Cells were then treated with 3 µg/mL LPS diluted in culture medium for 6 h. PEA (100 µM) was applied 1 h before LPS and maintained in contact with the cells throughout the whole LPS exposure. After the incubation period, total RNA was extracted from N9 cells using EuroGOLD Trifast reagent (Euroclone, Pero, Italy) according to the manufacturer’s instructions and quantified using Nanodrop ND-1000 spectrophotometer (Thermo Fisher Scientific, Waltham, MA, USA). Reverse transcription was performed using iScript cDNA Synthesis Kit (Bio-Rad, Hercules, CA, USA). Real-time PCR (RT-PCR) was carried out on a QuantStudio 7 Flex Real-Time PCR System (Applied Biosystem, Foster City, CA, USA) using the iTaq Universal Probes Supermix (Bio-Rad, Hercules, CA, USA). Pairs of primers and Taqman probes (Taqman Gene Expression Assays) were obtained from Applied Biosystem (Foster City, CA, USA). RT-PCR was performed as follows: 50 °C for 2 min, followed by 95 °C for 10 min, and lastly 40 cycles at 95 °C for 15 s and 60 °C for 1 min. Relative mRNA concentrations of the target genes were normalized to the corresponding β-actin internal control and calculated using the 2^−ΔΔCt^ method. Data were analyzed using GraphPad v6.0 software (San Diego, CA, USA), employing ANOVA, followed by Tukey’s test for group comparison. *p* < 0.05 was considered statistically significant.

### 4.4. Western Blot Analysis

N9 cells were plated in 6-well culture plates at a density of 35 × 10^4^ cells/well, incubated at 37 °C for 48 h, and then treated as indicated on PCR analysis; for the experiments with selective inverse agonist of CB2, cells were incubated with 100 nM SR144528 1 h before the treatment with PEA and LPS. After incubation, cells were collected and lysed in radioimmunoprecipitation assay (RIPA) buffer (Cell Signaling Technology, Danvers, MA, USA). The total protein concentration was determined using the Pierce BCA Protein Assay Kit (Thermo Fisher Scientific, Waltham, MA, USA). Equal amounts of proteins were heated at 95 °C for 10 min, loaded on precast 4–12% gradient gels (Twin Helix, Rho, Italy), separated by electrophoresis, and transferred to a polyvinylidene difluoride (PVDF) membrane (Thermo Fisher Scientific, Waltham, MA, USA). Non-specific binding was blocked with 5% dried fat-free milk dissolved in phosphate-buffered saline (PBS) supplemented with 0.1% Tween-20 (PBS-T) for 1 h at room temperature (RT). After washes in PBS-T, membranes were incubated with the primary antibody overnight at 4 °C and then with a peroxidase-coupled secondary antibody for 1 h at RT. Blots were probed with anti-IL-1β (#31202, Cell Signaling Technology, Danvers, MA, USA, 1:1000), anti-iNOS (#13120, Cell Signaling Technology, Danvers, MA, USA, 1:1000) and anti-Arg1 (#93668, Cell Signaling Technology, Danvers, MA, USA, 1:1000). Rabbit primary anti-GAPDH antibody (#2118, Cell Signaling Technology, Danvers, MA, USA, 1:3000) was used for normalization. Peroxidase-coupled goat anti-rabbit IgG (#31460, Thermo Fisher Scientific, Waltham, MA, USA, 1:5000) was used as secondary antibody. Signals were developed with the Extra Sensitive Chemiluminescent Substrate LiteAblot TURBO (Euroclone, Pero, Italy) and detected with the Amersham ImageQuant 800 Western blot imaging system (GE Healthcare, Chicago, IL, USA). Image J software (National Institutes of Health, Bethesda, MD, USA) was used to quantify protein bands. For iNOS expression, in some of the blots only, a band of poor intensity was present in DMSO sample; however, it was possible to quantify it and to express the protein level of LPS and PEA group in terms of relative expression. The experiments were repeated three independent times. Data were analyzed using GraphPad v6.0 software (San Diego, CA, USA) employing ANOVA followed by Tukey’s test or by Kruskal–Wallis test and Dunn’s multiple comparisons test for group comparison. *p* < 0.05 was considered statistically significant.

### 4.5. Immunofluorescence

N9 cells were seeded at a density of 1 × 10^5^ cells/mL on porcine gelatin pre-treated coverslips. One day after seeding, cells were treated with 3 µg/mL LPS diluted in culture medium for 6 h. PEA (100 µM) was applied 1 h before LPS and maintained in contact with the cells throughout the whole LPS exposure. Cells were then stained with DiI (Sigma-Aldrich, St. Louis, MO, USA) to label cell membranes (according to the manufacturer’s instructions) and fixed for 10 min with 3.7% paraformaldehyde in phosphate-buffered saline (PBS). Fluorescence images were captured with a Leica TCSSP2 confocal microscope (Wetzlar, Germany) equipped with a 63×/1.4 NA Plan-Apochromat oil immersion objective.

### 4.6. Morphological Analysis

Morphological analysis (skeleton, fractal analysis and hierarchical cluster analysis) were performed following procedures previously described by Fernández-Arjona [51] and Morrison [52]. Figures S2 and S3 illustrate the whole process, applied on a representative image for skeleton and fractal analysis, respectively. Parameters that we decided to evaluate in this work were summarized in Table S1. Regarding hierarchical cluster analysis (HCA), we decided to consider all parameters calculated with FracLac analysis even if multimodality index (MMI) was not higher than 0.55. Data were analyzed using GraphPad v6.0 software (San Diego, CA, USA) employing ANOVA followed by Tukey’s test for group comparison. *p* < 0.05 was considered statistically significant.

### 4.7. Intracellular Calcium Mobilization Assay

Primary microglia cells and N9 cells were plated, respectively, at 50.000 and 20.000 cells/well into black walled, clear bottom 96-well plate (Greiner Bio One, Kremsmünster, Austria) and cultured up to 90–100% of confluence. Prior to assay, cells were incubated in dark conditions with 100 µL of Hank’s Balanced Salt Solution (HBSS) containing 20 mM HEPES, 2.5 mM probenecid and 4.5 µM FLUO-4 NW (Molecular Probes, Eugene, OR, USA) at 37 °C and 5% CO_2_ for 40 min. Fluorescence emissions were monitored with the multilabel spectrophotometer VICTOR^3^ (Perkin Elmer, MA, USA) at 485/535 nm (excitation/emission filters) every 0.5 s for the 20 s preceding and the 60 s following the stimulation. Changes in fluorescence corresponded to changes in intracellular calcium levels. ATP was diluted in HBSS solution and injected into the wells by an automated injector system. Where indicated, PEA was added to the cells 1 h before the injection with the stimulus. All experiments were performed at 37 °C, and fluorescence values (F) were normalized against the baseline acquired immediately before stimulation (F0).

### 4.8. Electrophysiological Recordings by MEA System

Primary cortical neuron/astrocyte/microglia cultures were prepared as previously described [53] in MEA Petri dishes pre-coated with polyethyleneimine 0.1% (*w*/*v*, Sigma-Aldrich, St. Louis, MO, USA). After 3 h incubation, the plating medium was replaced by neurobasal medium (NB) with B27, 10 ng/mL basic fibroblast growth factor (bFGF) (all Thermo Fisher Scientific, Waltham, MA, USA), and 1 mM glutamine (Sigma-Aldrich, St. Louis, MO, USA). Cultures were covered with gas-permeable covers (MEA_MEM, Ala Scientific Instruments, Inc., Farmingdale, NY, USA) and were maintained for 12–22 days at 37 °C in a humidified atmosphere with 5% CO_2_. Half of the medium volume was replaced every 3 days. Electrophysiological recordings were carried out at 12 to 16 days in vitro (DIV). Each MEA dish had a recording area of ~2 mm × 2 mm, constituted by 30 µm diameter ITO electrodes spaced 200 µm apart (Multichannels System, Reutlingen, Germany). In this area, the average number of neurons was ~6000, and astrocytes were about the same number. Drugs were added in volumes that were always < 1% of the total medium volume bathing the culture. As previously described [53,54], analog signals were recorded from the cultures at 36 °C in CO_2_-controlled incubator by using MEA 1060BC or 1060INV pre-amplifiers (bandwidth 1–8000 Hz, Multichannel Systems, Reutlingen, Germany) connected to a MEA Work-station (bandwidth 100–8000 Hz, Plexon Inc., Dallas, TX, USA). Data were sorted as described in Gullo et al. [18,19]. Since our previous studies demonstrated that LPS causes the appearance of atypical events in the network, characterized by bursts with long duration, in this paper the burst duration (BD), and more precisely the value of BD at 95% of the cumulative distributions of BD (cumBD95), was used as the representative parameter of an overall increase in neuronal excitability. CumBD of the network was computed as previously described [18,19]. Briefly, a running window of duration from 10 ms to 1 s was applied to search for the start and the end of bursts. Each burst and each spike in the burst were precisely assigned to the neuron firing them. For each burst, the average time course (from its start to its end) was calculated, and to investigate the heterogeneity of the BD, we studied its distribution in the form of cumulative probabilities (cumBD) in each time of interest by performing a standard cumulative probability analysis. Data were analyzed and the figures were prepared using Neuroexplorer (v4.133, Plexon Inc., Dallas, TX, USA) (Figure 9A) and OriginPro 2018 (v9.5.193, OriginLab Corporation, Northampton, MA, USA) (Figure 9B). Data in Figure 9C are expressed as mean ± S.E.M. Normality of data were tested with GraphPad Prism (v6.0, San Diego, CA, USA), and statistical significance of the difference of cumBD in LPS and in LPS after pretreatment with PEA was determined with multiple *t*-test corrected for multiple comparisons by using the Holm–Sidak method (GraphPad Prism v6.0).

### 4.9. TNF-α and BDNF Concentration Measurements

N9 cells were seeded at the concentration of 9 × 10^4^ cells/well into a 12-well plate and then the day after were treated as described above. Levels of TNF-α released into the culture medium were quantified after 6 h by using the corresponding quantification ELISA kits (BMS607, Thermo Fisher Scientific, Waltham, MA, USA). In each MEA dish, small (150 μL) aliquots of the incubation medium were collected in control and at 6 h (representing the peak-time of LPS-induced TNF-α release [18]) after the addition of LPS (3 μg/mL) or PEA + LPS or SR144528 + PEA + LPS. Samples (50 μL) were analyzed in triplicate for murine TNF-α and BDNF with ELISA kits (BMS607, Thermo Fisher Scientific, Waltham, MA, USA) (KA0331 Abnova, Walnut, CA, USA) according to manufacturer’s instructions. The data were expressed as pg/mL following interpolation on the basis of a standard curve. The experiments were repeated three independent times. Data were analyzed using GraphPad 6.0 software (San Diego, CA, USA) employing ANOVA followed by Tukey’s test for group comparison. *p* < 0.05 was considered statistically significant.

### 4.10. SEAP Assay

THP-1 X-Blue and HEK-Blue hTLR4 cells were specifically designed for monitoring the NF-κB signal transduction pathway. Respectively, 8 × 10^4^ and 3 × 10^4^ cells/per well were seeded into a 96-well plate. Cells were then treated with 10 ng/mL LPS diluted in culture medium. PEA (100 µM) was applied 1 h before LPS and maintained in contact with the cells throughout the whole LPS exposure. Levels of SEAP in the supernatant were easily determined after 6 h with Quanti-Blue solution according to manufacturer’s instructions (InvivoGen, San Diego, CA, USA). Activity of SEAP, expressed as OD, was normalized on value obtained by the control sample. Data was analyzed using GraphPad v6.0 software (San Diego, CA, USA) employing ANOVA followed by Tukey’s test for group comparison. *p* < 0.05 was considered statistically significant.

## Data Availability

Not applicable.

## Supplementary Materials

The following are available online at https://www.mdpi.com/1422-0067/22/6/3054/s1, Figure S1: Effect of PEAon Arg1 expression, Figure S2: Skeleton analysis of N9 microglia morphologies, Figure S3: FracLac for ImageJ to quantify cell complexity and shape, Table S1: Summary of microglia morphology parameters.
