# Supplementary material for: Palmitoylethanolamide Modulation of Microglia Activation: Characterization of Mechanisms of Action and Implication for Its Neuroprotective Effects
Source: Int J Mol Sci. 2021 Mar 17;22(6):3054. doi: 10.3390/ijms22063054 (PMC8002502; doi:10.3390/ijms22063054)
Supplement: Supplementary file 1 [file ijms-22-03054-s001.zip › Supplementary Figures.docx]

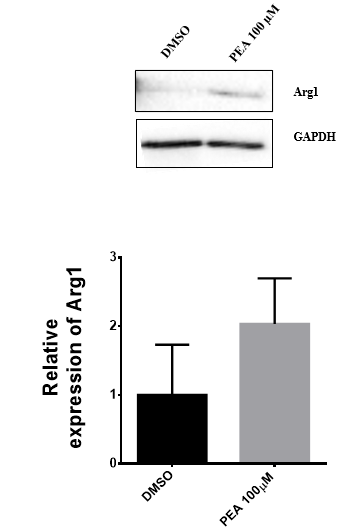


**Figure S1** *Effects of PEA on Arg1 expression****.*** N9 cells were incubated with 100 µM PEA for 6h. Total protein extract from N9 cells were separated on SDS-PAGE and transferred to a polyvinylidene difluoride (PVDF) membrane; membranes were probed with anti-Arg1 and anti-GAPDH. The panel shows an exemplificative western blot and the graphical representation of Arg1 expression. GAPDH was used to normalize sample loading. Data are shown as the mean ± SEM of measurements obtained in three independent experiments (n = 3). Differences among groups were tested for significance by the one-way analysis of variance (ANOVA) followed by Kruskal-Wallis test


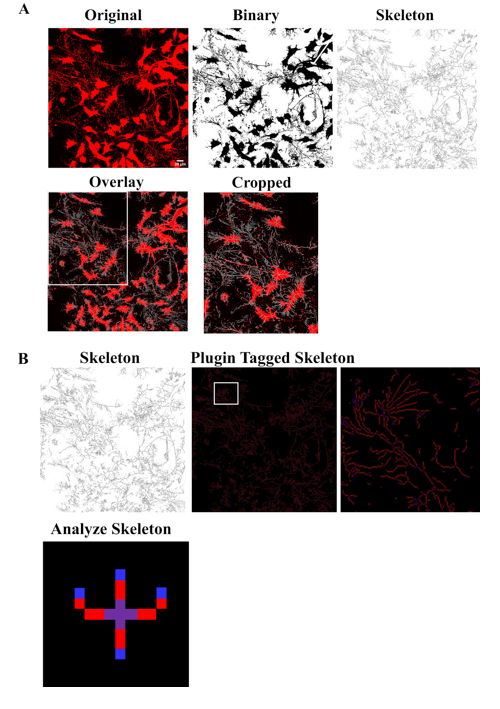


**Figure S2** *Skeleton analysis of N9 microglia morphologies.* (A) The process to prepare confocal photomicrographs for skeleton analysis. Original confocal images were subjected to a series of uniform ImageJ plugin protocols prior to conversion to binary images; binary images were then skeletonized. An overlay of a resulting skeletonized image (in cyan) and original photomicrograph shows the relationship between skeleton and photomicrograph. Cropped photomicrographs show additional details. Scale bar: 20µm. (B) The skeletonized images are processed using the Analyze Skeleton plugin (maintained here: http://imagej.net/AnalyzeSkeleton) to identify and tag skeletonized processes as orange, endpoints as blue, and junctions as purple. The tagged data are then organized, and data output summarized.


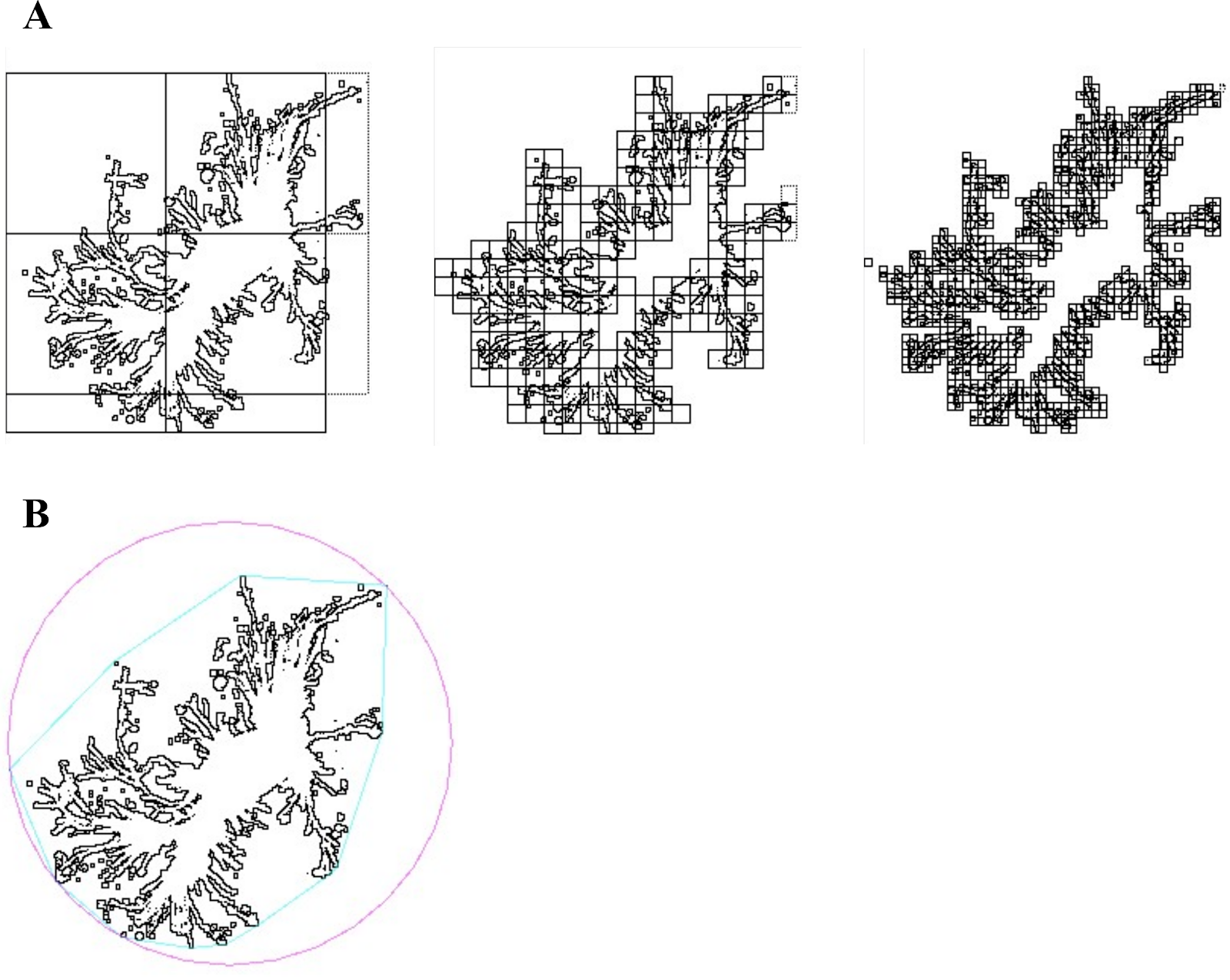


**Figure S3** *FracLac for ImageJ to quantify cell complexity and shape.* (A) Illustration of FracLac box counting method to derive fractal dimension calculations of a microglia outline. Shape detail is quantified as scale increases, represented by black boxes. Box counting equation is summarized in Table S1. (B) Schematic of the convex hull (cyan) and convex hull ellipse (pink) necessary for calculating microglia span ratio and density (Table S1).
