# Supplementary material for: Palmitoylethanolamide Modulation of Microglia Activation: Characterization of Mechanisms of Action and Implication for Its Neuroprotective Effects
Source: Int J Mol Sci. 2021 Mar 17;22(6):3054. doi: 10.3390/ijms22063054 (PMC8002502; doi:10.3390/ijms22063054)
Supplement: Supplementary file 1 [file ijms-22-03054-s001.zip › TableS1.pdf]

Table S1 Summary of microglia morphology parameters (Skeleton and FracLac analyses)

	Measure	Unit	Scale	Interpretation
Process lenght	Summed	µm/cell	Photomicro-graph	Cell ramification
Process endpoints	Summed	#/cell	Photomicro-graph	Cell ramification
Fractal Dimension	Regression slope $\left[\frac{In\ (N)}{In\ (\varepsilon)}\right]$	D_B	Individual cell	Cell complexity
Span Ratio	$\frac{convex\ hull\ eclipse\ longest\ lenght}{convex\ hull\ eclipse\ longest\ width}$	Ratio	Individual cell	Cell shape
Density	$\frac{\#\ of\ pixels\ within\ cell\ outline}{area\ of\ convex\ hull}$	$\frac{\#\ of\ pixels}{area}$	Individual cell	Cell size
Area	Total number of pixels present in the filled shape of cell image	µm^2	Individual cell	Cell size
Perimeter	# of pixels expressed in microns	µm	Individual cell	Cell size
Lacunarity	heterogeneity or translational and rotational invariance in a shape	mass distribution of pixels	Individual cell	Shape changing
Circularity	$\frac{(4\pi \times cell\ area)}{(cell\ perimeter)^2}$	Ratio	Individual cell	Cell shape
